# Emerging Nonpharmacologic Analgesic Technologies in Anesthesia: Mechanisms, Evidence, and Future Directions for Pharmacologic Alternatives

**DOI:** 10.3390/biomedicines14010225

**Published:** 2026-01-20

**Authors:** Alyssa McKenzie, Rachel Dombrower, Sophia McKenzie, Nitchanan Theeraphapphong, Alaa Abd-Elsayed

**Affiliations:** 1 School of Medicine, St. Georges University, University Centre Grenada, West Indies 11739, Grenada; racheldombrower@gmail.com (R.D.); ntheerap09@gmail.com (N.T.); 2 School of Medicine, Uniformed Services University of the Health Sciences, Bethesda, MD 20814, USA; sophia.mckenzie@usuhs.edu; 3 Department of Anesthesiology, University of Wisconsin School of Medicine and Public Health, Madison, WI 53726, USA; abdelsayed@wisc.edu

**Keywords:** nonpharmacologic analgesia, neuromodulation, peripheral nerve stimulation, spinal cord stimulation, opioid-sparing anesthesia, device-based analgesia, neuroimmune modulation, opioid alternatives

## Abstract

Perioperative pain remains a major clinical challenge, with many surgical patients experiencing inadequate analgesia and progression to chronic postsurgical pain. Conventional opioid-centered strategies are limited by narrow therapeutic windows, systemic toxicity, tolerance, opioid-induced hyperalgesia, and poor efficacy in neuroimmune-driven pain states. Advances in molecular neuroscience and biomedical engineering have catalyzed the development of nonpharmacologic analgesic technologies that modulate pain pathways through biophysical rather than receptor–ligand mechanisms. This narrative review synthesizes emerging nonpharmacologic analgesic platforms relevant to anesthesiology, integrating molecular, cellular, and systems-level mechanisms with clinical evidence. It examines how peripheral sensitization, spinal dorsal horn plasticity, glial and neuroimmune activation, and supraspinal network dysfunction create ideal targets for device-based interventions. Electrical neuromodulation strategies, including peripheral and central techniques, are discussed alongside temperature-based, photonic, and focused-energy modalities. These include cryoneurolysis, radiofrequency techniques, photobiomodulation, and low-intensity focused ultrasound. Clinical integration within enhanced recovery pathways, patient selection, workflow considerations, and limitations of the current human evidence base are reviewed. While many of these technologies are established in chronic pain management, this review emphasizes available human perioperative data and discusses how chronic pain evidence informs perioperative translation within opioid-sparing multimodal anesthesia care. Collectively, these technologies support a mechanism-based, systems-level approach to pain modulation, with perioperative relevance varying by modality and strength of available human evidence.

## 1. Introduction

Pain management during the perioperative and postoperative periods is a significant concern for anesthesia professionals. More than 80% of patients experience moderate-to-severe postoperative pain following surgery. Approximately 20–30% of patients develop chronic postoperative pain [1,2]. Pain may arise from complex processes involving peripheral nociceptors, dorsal horn neurons, and central immune system activation facilitated by tissue injury from surgical procedures and pre-existing chronic pain [3,4,5].

Though opioids are considered prominent analgesics in perioperative care, they are increasingly used within multimodal strategies due to their narrow safety therapeutic window and considerable physiologic liabilities [6,7]. Clinically relevant adverse effects include tolerance, opioid-induced hyperalgesia (OIH), dependence, gastrointestinal dysmotility, and respiratory depression, all of which complicate perioperative management [6,7,8]. Beyond these limitations, opioid-based therapies do not target core molecular processes responsible for acute and chronic pain, including glial activation, dysregulated glutamatergic neurotransmission, and maladaptive neuroimmune signaling [5,6]. In contemporary perioperative practice, opioid-sparing strategies also include regional and infiltrative analgesia techniques, multimodal pharmacologic adjuncts, and physiological guidance of analgesic delivery, all of which contribute to improved postoperative pain control and may reduce the risk of chronic postsurgical pain.

These drawbacks highlight the need for mechanism-based, nonpharmacological approaches to pain management that target nociceptive pathways without the systemic toxicity associated with conventional pharmacological therapies [6,7,8]. Many nonpharmacologic analgesic technologies were developed and validated in chronic pain populations, and their perioperative relevance varies based on available human data, procedure type, and timing of intervention.

Current approaches to pain management reflect a shift towards precision analgesia [9]. Molecular neuroscience research has established the importance of microglia, satellite glial cells, and neuroimmune signaling interactions in the development and persistence of pain [10,11]. These findings have renewed interest in developing more modern approaches for treating pain through electrical and biochemical signaling at specific sites [10].

Recent advances in biomedical engineering have enabled the integration of device-based therapies with simultaneous physiological feedback. This has facilitated the development of feedback-based therapies [12]. Closed-loop neuromodulation, focused ultrasound, photobiomodulation, and energy-based therapies can now be applied to peripheral and central nervous system circuits with unprecedented precision [12]. Nonpharmacologic analgesic platforms are increasingly incorporated into perioperative care as adjunctive components of multimodal analgesia, with adoption varying by procedure, setting, and strength of human evidence.

This review provides a mechanistic, systems-level integration of nonpharmacologic approaches to analgesia relevant to anesthesiology, with emphasis on human perioperative evidence where available. Interventions targeting peripheral, spinal, and supraspinal pathways are examined alongside glia-neuroimmune mechanisms, with chronic pain data discussed in the context of perioperative translation rather than direct acute substitution.

## 2. Molecular and Cellular Foundations of Pain Relevant to Nonpharmacologic Therapies

### 2.1. Peripheral Sensitization and Nociceptor Excitability

**Figure 1:**
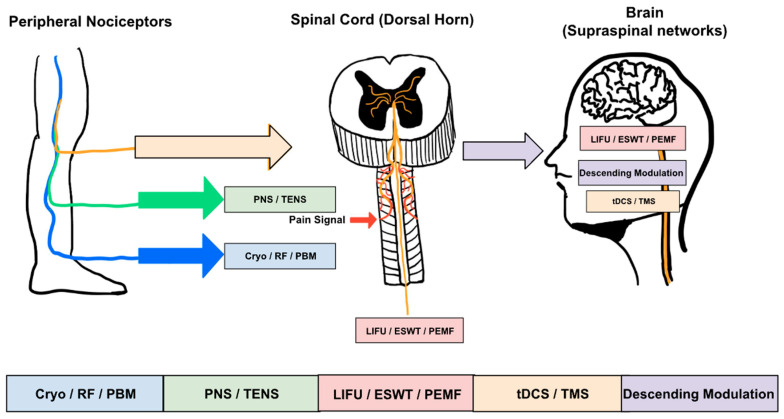
Systems-level pain pathways targeted by nonpharmacologic analgesic technologies. The arrows in Figure 1 indicate the direction of nociceptive signal transmission and the anatomical level at which each nonpharmacologic modality primarily acts. Arrows from the periphery to the spinal cord represent ascending nociceptive input, while arrows directed toward the spinal cord or brain indicate sites of therapeutic intervention that modulate pain processing at peripheral, spinal, or supraspinal levels.

Surgical damage and accompanying inflammation trigger a cascade of molecular interactions at the nerve terminals, increasing the excitability of nociceptors [13]. The release of various mediators, including prostaglandins, bradykinin, ATP, nerve growth factor, and the pro-inflammatory cytokines IL-1β and TNF-alpha, accompanies tissue injury. These mediators increase the excitability of nociceptors by altering the functions of ion channels [14]. Ion channels, such as TRPV1, Nav1.7, Nav1.8, and pH-sensitive channels, undergo phosphorylation and upregulation, which lowers activation thresholds and enhances afferent excitability [14]. Altered ion channel function and membrane excitability are accessible targets for device-based therapies, such as transcutaneous electrical nerve stimulation, percutaneous peripheral nerve stimulation, cryoanalgesia, focused ultrasound, and photobiomodulation [15]. These interventions result in the suppression of nociceptor ectopic activity, decreased production of inflammatory mediates, and altered membrane excitability [15,16]. As a result, peripheral tissues become amenable to device-based modulation to produce analgesia. These interactions occur across peripheral, spinal, and supraspinal levels of the pain pathway, which are schematically illustrated in Figure 1.

### 2.2. Spinal Dorsal Horn Processing and Central Sensitization

The dorsal horn of the spinal cord undergoes substantial neurophysiological plasticity following chronic exposure to nociceptive stimuli [17,18]. Excitatory transmission is enhanced by increased NMDA receptor activity and calcium-permeable AMPA receptors, while inhibitory control is reduced through loss of GABAergic and glycinergic interneurons [18,19]. These mechanisms are involved in the pathogenesis of central sensitization, characterized by hyperalgesia, allodynia, and enhanced temporal summation of pain [20]. Structural plasticity, including the abnormal extension of Aβ low-threshold afferents targeting nociceptive spinal layers, further disrupts sensory integration [18].

Dorsal horn excitability can be adjusted using nonpharmacologic technologies that shift the excitation-inhibition balance [21]. Peripheral nerve stimulation, spinal electrical stimulation, pulsed radiofrequency, and focused ultrasound can alter interneuronal circuits, reduce pathologic neurotransmitter release, and counter dorsal horn hyperexcitability [22]. Device-based approaches can provide relief in chronic nociplastic pain states by decreasing central sensitization.

### 2.3. Neuroimmune Activation and the Role of Glial Cells

Glial cells are a critical component of acute and chronic pain circuits [23]. Microglia respond specifically to peripheral damage via the P2X4, P2X7, TLR4, and BDNF signaling pathways. This response triggers the expression of IL-6, IL-18, TNF-α, and other mediators that disrupt chloride homeostasis and inhibitory transmission [24]. Simultaneous astrocyte activation promotes excitatory neurotransmitter release, thereby sustaining a pro-inflammatory, hyperexcitable neural environment [25]. Satellite glia within the dorsal root ganglia further maintain this neuroimmune dysfunction by enhancing intercellular and extracellular signaling [26]. Various nonpharmacological analgesic therapies can selectively alter this neuroimmune function. For instance, photobiomodulation and focused ultrasound have been shown to downregulate microglial activation and associated signaling, modulate cytokine release, and influence glial-mediated regulation of neurotransmission, producing sustained anti-inflammatory effects within pain-related glial-neuronal networks [27,28,29].

### 2.4. Supraspinal Modulation and Descending Inhibitory Control

Pain integration at the supraspinal centers is complex and is often underrecognized in pain management. The periaqueductal gray, rostral ventromedial medulla, thalamus, and cortical regions, including the somatosensory cortex, insula, and anterior cingulate cortex, integrate the sensory and affective components of pain [30,31]. Dysfunction of the descending inhibitory pathways, primarily serotonergic and noradrenergic pathways, is associated with reduced endogenous antinociception and increased pain hypersensitivity [30,32]. Noninvasive brain stimulation methods, including transcranial magnetic stimulation and transcranial direct and alternating current stimulation, can influence cortical excitability and increase the strength of descending inhibitory circuits [30,32]. Advances in focused ultrasound have enabled more precise stimulation of subcortical regions [33]. These approaches modulate pain by rebalancing higher-order networks, an effect that pharmacotherapy achieves only indirectly.

### 2.5. Connecting Molecular Mechanisms to Nonpharmacologic Analgesic Technologies

Peripheral sensitization, spinal plasticity, neuroimmune activation, and supraspinal dysfunction collectively drive acute and chronic pain [34,35]. Nonpharmacological methods for pain relief interact with these pathways through biophysical interactions with ion channels, neurochemical release, activation of the inflammation cascade, glial signaling, and neural network oscillations [29,35]. These approaches operate outside classical receptor-ligand pharmacology and avoid key limitations of systemic analgesics [6,35]. Advances in engineering have established these technologies as efficient options for perioperative and chronic pain management [29,35].

## 3. Principles of Nonpharmacologic Analgesic Technologies

### 3.1. Mechanistic Foundations: Biophysical Modulation of Neural and Immune Pathways

**Figure 2:**
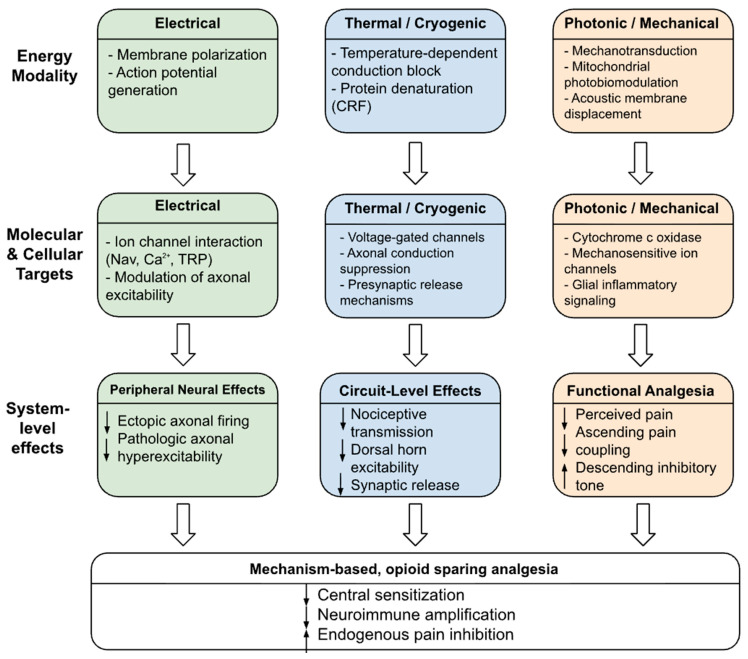
Electrical, thermal/cryogenic, and photonic/mechanical energy modalities engage distinct molecular and circuit-level mechanisms to suppress nociceptive signaling and enhance endogenous inhibition, resulting in mechanism-based, opioid-sparing analgesia. The vertical arrows depict a conceptual progression from energy modality to molecular and cellular targets, followed by system-level effects that culminate in functional analgesia and opioid-sparing outcomes.

Nonpharmacological approaches to pain management utilize various biophysical principles to target nociceptive signaling across pain-processing pathways [16,21,36]. They involve the use of electrical, magnetic, thermal, optical, and mechanical energy to increase the excitability of neural tissues and affect ion channels and neuroimmune interactions [33,36,37]. This contrasts with systemic pharmacotherapies, which depend on ligand–receptor interactions. The transcutaneous, percutaneous, and focused energy field approaches used for treatment delivery affect membrane potential through processes associated with ectopic firing and sensitization of the peripheral and central nervous systems [22,35,36]. Their ability to impact processes in both neurons and glia supports the use of device-based strategies in clinical practice. The hierarchical relationships between energy modality, molecular and cellular targets, and system-level analgesic effects are summarized in Figure 2.

### 3.2. Target Specificity and Graded Invasiveness

Technological advances in nonpharmacologic analgesia are centered around targeting specific anatomical locations across a spectrum of precision and invasiveness, enabling patient-tailored pain modulation [21]. Peripheral approaches target more superficial nerves by dampening nociceptive signaling at the source, limiting the transmission of pain signals to the spinal dorsal horn [16]. Central approaches target more complex structures within the cerebral cortex and subcortical areas [21,32]. Energy-based therapies enable noninvasive modulation of deep neural targets with greater anatomical precision than surgically implanted devices, influencing both neural conduction and local inflammatory processes [27,28]. This supports the adoption of personalized approaches across acute and chronic pain states.

### 3.3. Modulation of Neuroimmune and Glial Activity

The involvement of neurons, glia, and immune cells in pain management has been increasingly reported [38,39,40]. Several methods for managing pain without medication involve inhibiting glial inflammation by reducing pro-inflammatory cytokines and correcting synaptic function [27,38,39]. Electrical neuromodulation can inhibit the activation of immune cells by glia through modulation of the dorsal horn neural firing rate [27,29,41]. Focused ultrasound and pulsed energy therapies disrupt neuroimmune feedback loops by interrupting mechanosensitive ion channels and inflammatory signaling [9,19,28].

### 3.4. Integration with Systems-Level Pain Networks

Pain perception arises from the integration of peripheral signaling, spinal cord circuits, and complex supra-spinal networks [21,29,35,42]. Nonpharmacological approaches can intervene at distinct levels within this integrated pain network [21,35]. Peripheral approaches downregulate nociceptor signaling and reduce dorsal horn excitability [17,36], whereas central approaches act more directly on cortical oscillatory processes and higher-order networks involved in emotion and executive function related to pain [32,43]. Energy-based approaches may offer additional support for system-level interactions by reducing local inflammation and limiting chronic nociceptive drive [28,37]. Together, these nonpharmacologic approaches create a directed systems-level analgesic strategy consistent with the biopsychosocial and neuroimmune models of pain.

### 3.5. Clinical Rationale: Toward Precision, Opioid-Sparing Analgesia

Nonpharmacologic pain strategies are increasingly relevant in contemporary anesthesia practice. These strategies represent a legitimate alternative to opioid-based treatment therapies, particularly because of their ability to precisely target the nociceptive process at various biological levels, including ion channels, glia, and cortex [21,29,35,44,45]. Their favorable safety profiles, lack of systemic toxicity, and compatibility with enhanced recovery support their incorporation into modern perioperative and postoperative care [44,45,46,47]. As engineering innovations improve device precision and translational research refines target specificity, nonpharmacologic modalities are likely to become integral components of precision, opioid-sparing anesthesia. Table 1 provides a comparative overview of nonpharmacologic analgesic technologies, summarizing their primary targets, mechanisms, typical indications, and perioperative use cases across acute and chronic pain contexts.

## 4. Electrical Neuromodulation Platforms in Anesthesia

### 4.1. Peripheral Nerve Stimulation (PNS)

Peripheral nerve stimulation (PNS) involves the delivery of precisely controlled electrical currents to specific peripheral nerves, influencing nociceptive feedback before it is transmitted to the spinal cord [62,63,64]. This can be accomplished via open-loop percutaneous PNS, in which stimulation parameters remain unchanged and unmodified based on physiologically measurable feedback [65,66,67]. Pain relief is achieved by activating Aβ afferents, promoting sensory gating within the spine, and reducing ectopic neural firing from irritated nerves [62,63,67].

PNS has specific neuroimmunomodulatory effects, including reduced production of pro-inflammatory cytokines and diminished activation of microglia and satellite glia [63,68,69,70]. Its percutaneous administration has been associated with reduced postoperative pain, reduced opioid consumption, and increased durable relief from focal neuropathic symptoms, such as post-amputation pain and postoperative neuralgia [63,65,66]. These findings demonstrate the potential of PNS as a minimally invasive therapy and adjunct for perioperative pain management.

### 4.2. Central Neuromodulation (tDCS, tACS, TMS)

Central neuromodulatory methods, such as transcranial direct current stimulation (tDCS), transcranial alternating current stimulation (tACS), and transcranial magnetic stimulation (TMS), target the cortical and subcortical circuits involved in pain perception [21,32,49,71]. They can rebalance the excitation and inhibition levels within the cortex and modulate the sensory, affective, and cognitive aspects of pain perception by altering the excitability of neurons and synchronizing their oscillations [21,49].

Central neuromodulation enhances descending inhibitory pathways originating from prefrontal and motor areas, thereby potentiating brainstem-mediated suppression of dorsal horn activity [21,72]. These circuit modifications are accompanied by decreases in neurotransmission and cortical neuroinflammation [21,29]. Emerging evidence suggests that central modulation may be a treatment option for postoperative pain, as well as central sensitization, as a complementary treatment to peripheral and spinal interventions [21,49].

### 4.3. Closed-Loop PNS (Adaptive Neurostimulation)

Closed-loop PNS represents an advanced evolution of technology from traditional open-loop platforms, incorporating real-time physiological feedback to adjust stimulation delivery [73,74,75] dynamically. Signals such as evoked compound action potentials (ECAP) are measured and used to modulate the device’s stimulation parameters, including amplitude, pulse width, frequency, and timing [73,74,75]. Rather than relying on fixed preset settings, closed-loop PNS can administer consistent neural activation despite marked variations in tissue electrical properties and neural excitability [73,74].

Adaptive stimulation is more effective at stabilizing afferent transmission, reducing abnormal neural activity, and enforcing inhibitory interactions within the spinal circuitry [75,76]. By inducing significant downstream effects, closed-loop PNS can reduce microglial activation and promote anti-inflammatory signaling [68,69]. Closed-loop PNS has excellent potential for precision dosing in dynamic physiological conditions.

### 4.4. Spinal Cord Stimulation—Relevance to Anesthesia

Spinal cord stimulation (SCS) is the earliest form of neuromodulation used for chronic pain, providing important translational insights for neuromodulation devices [21,77,78]. Conventional tonic SCS activates dorsal column Aβ fibers to induce paresthesia and inhibit nociceptive transmission. In contrast, paresthesia-free high-frequency SCS (HF10) exerts its effects through non-parasthesia mechanisms, involving the modification of wide dynamic range neurons and the suppression of dorsal horn hyperexcitability [77,78,79,80]. Evidence suggests that HF10 provides more consistent analgesia with reduced posture-related variability compared to traditional SCS [77,78,81].

The progression of spinal cord stimulation from tonic waveforms to high-frequency and ECAP-based closed-loop systems underscores the importance of waveform specificity and physiologic feedback in managing chronic neuropathic pain [78,82]. These advancements have influenced the next generation of peripheral neuromodulation, which focuses on durable neural recruitment and minimizing overstimulation [78,82]. Optimized electrical dosing also reduces the risk of treatment-induced central sensitization.

### 4.5. Wearable Neuromodulation (TENS, Interferential Stimulation)

Transcutaneous electrical nerve stimulation (TENS) and interferential stimulation are portable approaches for the non-invasive treatment of nociceptive processing [16,35,48]. TENS selectively activates Aβ-afferent terminals to induce a spinal sensory gate effect and increase descending inhibitory controls by activating endogenous opioids and monoamines [35,83].

Interferential stimulation, in particular, employs intersecting current waves to generate more intense and lower-frequency fields with improved comfort and penetration [35,48]. Both approaches have been associated with significant relief from acute and postoperative pain, along with reduced opioid consumption [16,35]. Ease of transport and safety considerations make TENS and interferential stimulation essential additions to the recovery process and management.

## 5. Temperature-Based and Photonic Neuromodulation Techniques

Thermic and photonic neuromodulatory approaches utilize controlled heat or optical energy to treat peripheral nerve tissues by modulating nociceptive, inflammatory, and neuroimmune processes [84,85]. Unlike electrical neuromodulation, which primarily alters neuronal excitability, temperature, and photonic-based therapies interact with ion channels, extracellular matrix components, mitochondrial function, and immune signaling through distinct biophysical and biochemical mechanisms [85,86]. Their ability to disrupt pathogenesis at the cellular level enables localized modification of nerve tissues, supporting their use in perioperative and traumatic pain [50,87].

### 5.1. Cryoneurolysis

Cryoablation of nerves induces a temporary conduction block achieved through rapid cooling to temperatures of −60 °C to −100 °C, resulting in reversible Wallerian degeneration distal to the lesion while preserving the epineurial and perineurial layers [50,88,89]. Preservation of these connective tissue layers allows predictable axonal regeneration, with analgesic effects lasting from several weeks to months [88,89].

Cryoablation of nerves extinguishes nociceptor function by blocking conduction through the downregulation of sodium and potassium channels, thereby preventing the propagation of action potentials [50,88,90]. In the clinical setting, cryoneurolysis has been successful in managing postsurgical pain, such as knee arthroplasty and thoracic surgery, traumatic rib fractures, and entrapment neuropathies [51,52,91]. In the perioperative setting, it can assist with analgesia beyond the duration achieved with single-shot nerve blocks, reduce opioid usage, and serve as a practical alternative when continuous peripheral nerve catheters are not available [51,91,92]. Cryoneurolysis is particularly well-suited for procedures with intense nociceptive input [51,52,91].

### 5.2. Radiofrequency Modalities

Radiofrequency (RF) methods provide controlled current delivery to neural structures, achieving thermal or subthermal effects, depending on the delivery form [53,54]. Pulsed radiofrequency (PRF) ablation utilizes lower temperatures (<42 °C) to achieve neuromodulation without neurodestruction [53,54]. By changing synaptic transmission, PRF ablation downregulates inflammatory cytokines, such as TNF-alpha and IL-6, and alters the excitability of wide dynamic range neurons [54,55]. These changes may be associated with field effects that alter voltage-gated channels and intracellular signaling rather than nerve damage [53,54].

In contrast, conventional radiofrequency (CRF) uses temperatures of 60–90 °C to achieve thermal destruction of nerve tissue via ablation of nociceptive nerve fibers [56]. CRF is more suitable for chronic conditions, such as facetogenic back pain [93]. Because PRF preserves neural integrity while modulating inflammatory and excitability pathways, it is generally favored in settings where reversible analgesia is desired [53,55]. It is necessary to recognize the differences between PRF and CRF when applying them towards proper patient selection and timing in multimodal analgesic approaches.

### 5.3. Photobiomodulation and Laser-Based Analgesia

Photobiomodulation (PBM) utilizes low-intensity lasers or light-emitting diodes (LEDs) to deliver energy without heating tissues to levels that cause tissue ablation [94,95,96]. PBM activates cytochrome C oxidase and increases the concentrations of ATP and reactive oxygen species in the mitochondria [94,95]. It also reduces microglial activation, modulates glial inflammatory signaling, and facilitates the repair of peripheral nerves [27,95,97].

Clinically, PBM has been used to manage postoperative pain, tendon and joint injuries, neuropathic pain disorders, and oral and maxillofacial surgery [98,99,100]. The noninvasiveness of PBM and ease of implementation support its utility in settings prioritizing opioid minimization and accelerated recovery [94,98]. Ongoing advances in peak wavelength and tissue penetration may further expand the utility of PBM for acute pain management.

### 5.4. Hydrodissection and Perineural Interface Modulation

Hydrodissection involves injecting a liquid solution, either saline or a dilute form of local anesthesia, between fascial structures and perineural tissues to separate the nerves from other tissues [101,102]. Rather than relying on electrical neuromodulation, this technique primarily restores nerve mobility by relieving perineural adhesions and mechanical entrapment, reducing painful afferent signaling from inflamed nerves [101,102]. Expansion of the perineural interface may also improve local vascular perfusion and reduce inflammatory burden [101,103]. Perioperatively, administration of hydrodissection can facilitate accurate placement of the PNS lead for more effective electrical nerve modulation. It may also optimize the tissue environment for adjunctive thermal or energy-based interventions by improving perineural spacing and tissue compliance [101,102,104].

## 6. Focused Energy–Based Analgesic Technologies

Focused energy modalities represent a rapidly growing class of nonpharmacological analgesic methods that target nervous activity using precisely measured mechanical and electromagnetic waves [105,106]. Their effects are mediated by biophysical interactions with neuronal membranes and ion channel–associated signaling pathways, without the need for surgical incisions or implanted leads [105,107]. Given their capacity to access deep neural targets noninvasively, they hold particular promise for intraoperative and perioperative analgesia [105].

### 6.1. Low-Intensity Focused Ultrasound Neuromodulation

Low-intensity focused ultrasound (LIFU) involves the use of a tightly focused, low-intensity acoustic beam to modulate neural tissue without inducing cavitation or clinically significant thermal injury [108,109]. LIFU exerts its effects through interactions between neuronal membranes and mechanosensitive ionic channels, leading to alterations in intracellular calcium signaling and membrane excitability [109,110]. Depending on frequency and pulse parameters, LIFU can either inhibit or potentiate action potential firing [108,111]. Indirectly, preclinical data suggest that LIFU can inhibit microglial activation and alter astrocyte cytokine expression, thereby enhancing neuroimmune homeostasis [112,113].

LIFU serves as a novel approach for targeting central pain pathways, as evidenced by its ability to target deep structures such as the thalamus, insula, and brainstem nuclei [33,114,115]. Emerging perioperative applications include potential real-time analgesia during surgery, awake craniotomy procedures requiring rapid neurological assessment, and attenuation of surgery-associated central sensitization [116,117,118].

### 6.2. Other Focused and Mechanical Energy

Various other energy technologies provide neuromodulation of pain pathways through biophysical mechanisms, including tissue deformation, electromagnetic coupling, and audio field modifications. Although each may have distinct penetration depths and tissue interaction properties, many mechanical energy–based technologies share a common ability to alter nociceptive transmission without the need for systemic pharmacology [119,120].

Extracorporeal shockwave therapy (ESWT) utilizes high-intensity pressure waves to induce localized biomechanical stress in soft tissues [58,121]. It exerts its effects by targeting specific ion channels, interrupting peripheral nociceptive activity, and stimulating tissue regeneration through vascular endothelial growth factor (VEGF)-mediated angiogenesis and modulation of inflammatory cytokine concentrations [58,121]. Clinical data have shown ESWT to be effective in treating musculoskeletal pain disorders, tendinopathies, and neuropathic disorders. It has also shown promise for use in perioperative rehabilitation [59,60,122].

High-Intensity Focused Ultrasound (HIFU) focuses energy to generate a controlled thermal lesion in the target tissue and has been conventionally used for cancer ablation [123,124]. In analgesic settings, HIFU has been used for ablation of small peripheral nerve segments or manipulation of large musculoskeletal structures [123,124]. Its use in anesthesia for radiofrequency-free nerve ablation is emerging, although still in early trials.

Pulsed electromagnetic field therapy (PEMF) stimulates electromagnetic fields that affect transmembrane potentials, calcium oscillations, and inflammatory gene expression [125,126,127]. It has been used to reduce edema, influence macrophage polarization, and decrease pain signals generated via nociceptive neurons [127,128]. PEMF has been extensively used in rehabilitation medicine; however, it has recently garnered interest for use in perioperative services due to its potential benefits in wound healing and pain reduction, without the need for heat or electrical penetration [125,126,127,128].

## 7. Integrating Nonpharmacologic Technologies into Anesthesia and Perioperative Care

The successful use of nonpharmacological analgesic technologies depends on their structured incorporation into existing clinical protocols [45,129]. For instance, Enhanced Recovery After Surgery (ERAS) protocols depend primarily on factors such as appropriate patient selection and careful perioperative workflow management [45,130]. As evidence supporting nonpharmacologic analgesic strategies continues to accumulate, anesthesiologists are increasingly inclined to incorporate neuromodulation therapies alongside traditional pharmacologic approaches.

### 7.1. ERAS Protocols and Opioid-Sparing Anesthesia

Nonpharmacologic analgesic technologies integrate well with ERAS pathways by reducing opioid exposure and supporting postoperative recovery [1,131,132]. These approaches can be deployed preoperatively, intraoperatively, or postoperatively within multimodal analgesic pathways [1]. Representative interventions include peripheral nerve stimulation, cryoneurolysis, transcutaneous electrical nerve stimulation (TENS), photobiomodulation, and focused ultrasound.

Device-based therapies avoid adverse effects associated with pharmacologic adjuncts such as ketamine, gabapentinoids, and NSAIDs, including hemodynamic instability, renal dysfunction, and sedation risk [133,134,135]. The incorporation of neuromodulation into multimodal analgesia enables analgesic strategies to be tailored to the level of surgical invasiveness [1,131].

### 7.2. Patient Selection

The technologies should be applied to patients who are more likely to respond to the treatment provided by the device [136,137]. Neuropathic pain, complex pain syndromes, nerve injury, and surgeries associated with the risk of chronic postsurgical pain, namely thoracotomies, mastectomies, and joint replacements, may be effectively treated using PNS therapy, PRF, cryolysis, or photobiomodulation [50,66]. Pathologies with purely nociceptive, inflammatory, or deep musculoskeletal pain may benefit more from FUS therapy, SW, or RF therapies [58,121,138].

Emerging physiological biomarkers, including evoked compound action potential (ECAP) signatures, nerve conduction velocity parameters, and preoperative immunological profiles, may improve the accuracy of device selection for patients in the future [136,139]. Psychosocial and cognitive factors also influence treatment responsiveness and should be considered during patient selection [136,137].

### 7.3. Multisystem Considerations

Anesthesiologists must consider several multisystem implications before neuromodulation and energy-related therapies. Hemodynamic effects and implant interactions are significant considerations. Noninvasive therapies typically exert minimal systemic effects, even in patients with limited physiological reserve [140,141]. However, caution is required when implantable electrical devices are used in patients with other medical devices due to potential interference, such as those with cardiac pacemakers, spinal cord implant generators, or deep brain implant generators [141,142].

The risk profiles of cryoneurolysis, radiofrequency ablation, and focused ultrasound have been established in the literature. Cryoneurolysis may cause temporary sensory impairment, while radiofrequency ablation carries a risk of heat-induced tissue damage, and focused ultrasound requires real-time imaging to prevent extraparenchymal effects [65,93,143]. The relative contraindications for these procedures include infection at the intervention site, coagulopathy, malignancy along the intervention pathway, and severe peripheral neuropathy [65,93,144]. Despite their potential adverse effects, these neuromodulatory techniques generally exhibit a more favorable safety margin than systemic analgesics.

### 7.4. Subspecialty Applications

Nonpharmacologic analgesic technologies have been investigated across several anesthesia subspecialties, with perioperative evidence varying by procedure and pain mechanism. In orthopedic and regional anesthesia, peripheral nerve stimulation and cryoneurolysis have been studied following joint arthroplasty, rib fractures, and extremity surgery, primarily for postoperative pain reduction and opioid sparing [50,66,131]. In cardiothoracic and breast anesthesia, cryoneurolysis and nerve-targeted neuromodulation have been explored as adjuncts for thoracotomy- and mastectomy-related pain, particularly in patients at risk for chronic postsurgical pain [65,93,143]. Noninvasive modalities such as transcutaneous electrical nerve stimulation, photobiomodulation, and pulsed electromagnetic field therapy have been examined mainly in ambulatory, obstetric, and recovery-phase settings due to favorable safety profiles, though perioperative evidence remains limited [61,145]. Across subspecialties, these technologies are best positioned as adjuncts within multimodal analgesia frameworks rather than as standalone perioperative interventions.

### 7.5. Workflow & Implementation

The effective integration of nonpharmacologic pain management technologies depends on workflow design. Cryoneurolysis and PNS lead placement are performed preoperatively, while TENS, photobiomodulation therapy, and PEMF are administered during recovery [61,65,145]. Focused ultrasound therapy can be administered during surgery or postoperatively, depending on the target tissue [61,146].

Successful implementation requires collaboration between general anesthesiologists and pain management specialists to ensure appropriate case selection and management. Uniform protocols for drug administration, stimulation intensity, and follow-up studies would help limit the variability associated with treatment response [136,139].

## 8. Limitations of Current Evidence and Barriers to Adoption

Several factors hinder the adoption of nonpharmacologic approaches to pain management in perioperative care and complicate the interpretation of available data. Several therapies are still in the early stages, and although current data are encouraging, the evidence is insufficient to conclude efficacy and optimal perioperative implementation.

### 8.1. Limitations of the Current Evidence Base

Most device-based analgesic therapies have been evaluated in small-scale and feasibility studies [50,65]. However, some methods, such as transcutaneous electrical nerve stimulation and cryoneurolysis, have been validated through rigorous large-scale studies, including randomized controlled trials [147,148]. Few studies have aimed to validate endpoints related to the mechanisms of action, the prevention of chronic postsurgical pain, and chronic effects [66,149]. Most have reported subjective endpoints, such as pain measures, without using objective physiological or neuroimmune markers [62,66]. These limitations emphasize the need for standardized, purpose-designed clinical trials with clearly defined endpoints, many of which are now being addressed in ongoing and recently completed studies, as outlined in Section 8.5.

### 8.2. Variability in Stimulation Parameters and Protocols

A significant challenge for reproducibility is the lack of standardized dosing protocols for electrical, heat-based, photonic, and focused energy modalities. Parameters such as intensity, frequency, pulse width, thermodynamic properties, duration of treatment sessions, and target sites vary considerably among studies [57,150]. This often results in inconsistent clinical responses, limiting objective comparisons across studies. In peripheral nerve stimulation, photobiomodulation, and low-intensity focused ultrasound, small changes in device parameters can reverse biological effects or unintentionally shift treatments from neuromodulation to ablation [62,150,151].

### 8.3. Cost, Accessibility, and Training

These nonpharmacologic approaches reduce dependence on systemic analgesia. However, some may be costly due to the equipment and staff training required. Devices such as probes for cryoneurolysis and focused ultrasound systems, as well as those for percutaneous neuromodulation, may be unavailable in smaller centers and low-resource environments [152,153]. The need for specialized training and clinical experience may initially limit the safe and effective adoption of these technologies [139,140]. Without standardized compensation, adoption may remain uneven across clinical settings.

### 8.4. Need for Standardized Outcome Measurements

There are no consensus outcome measures for assessing analgesic response. Standard pain assessment tools provide useful information but do not capture end points such as glial activation levels, the strength of descending inhibition circuits, and measures of nociceptive signal profiles in the nervous system [154,155]. Standardizing outcomes, such as opioid-sparing response, time to mobilization after surgical intervention, indices of physiological nociception response, and biomarkers for neuroimmune interaction, would greatly facilitate inter-study comparisons. The current lack of standardized endpoints for studies has resulted in limited clinical interpretation.

### 8.5. Clinical Trials

In response to these recognized limitations, a growing number of clinical trials have sought to evaluate the safety, efficacy, and opioid-sparing potential of nonpharmacologic analgesic technologies across perioperative settings. These studies span a range of designs and stages, including completed, ongoing, and terminated studies. Completed and late-phase randomized trials have provided early evidence supporting the clinical applicability of peripheral neuromodulation strategies, including percutaneous peripheral nerve stimulation for postoperative pain and opioid reduction (NCT03481725, NCT03484429), as well as post-market evaluations of micro-implantable peripheral nerve stimulators (NCT05870124). Additional completed trials, such as the SNAP trial evaluating electrical nerve stimulation (NCT03783689), have provided further insight into the clinical applicability of this approach in perioperative and recovery settings.

Despite advancements, many studies remain limited by small sample sizes, heterogeneous stimulation parameters, and variability in perioperative measures. Several energy-based therapies, including wearable focused ultrasound systems (NCT07160049), are still in the early stages of clinical evaluation. Other trials have been terminated due to challenges such as patient enrollment difficulties, protocol complexity, and a lack of standardization (NCT03783689). Ongoing trials, including studies of accelerated central neuromodulation such as repetitive transcranial magnetic stimulation (NCT05295498), aim to address these gaps through improved trial design and incorporation of systems-level outcomes.

## 9. Future Directions—Emerging Strategies in Precision Analgesia

Advances in non-pharmacological analgesic technologies are driving the development of more precise and adaptive strategies for pain management. These developments have the potential to reshape how anesthesiologists assess, monitor, and manage nociception. The following sections outline key areas of future development.

### 9.1. Closed-Loop Anesthesia Systems Integrating Nociception Monitoring and Neuromodulation

Closed-loop analgesic systems integrate physiologic monitoring with automated delivery of nonpharmacologic neuromodulation. Advances in nociceptive monitoring permit continuous evaluation of autonomic markers of surgical pain, including the NOL index, pupillometry, skin conductance, and heart rate variability [156,157,158]. When combined with neuromodulatory technologies, these signals enable adjustment of stimulation based on variable nociceptive states. For example, techniques such as peripheral nerve stimulation may be adjusted based on evoked action potentials. Others, such as focused ultrasound and transcranial methods, can be adjusted based on cortical activity [158,159].

### 9.2. Bioelectronic Medicines and Electroceuticals

Bioelectronic medicine is a rapidly evolving field that extends neuromodulation to include the direct regulation of molecular pathways traditionally targeted by pharmacological therapies. While conventional electrical stimulation primarily modulates neural firing, newer electroceutical approaches are designed to influence neuroimmune circuits involved in cytokine signaling, macrophage activity, and inflammatory reflexes [160,161]. Targeted stimulation of specific pathways, such as the vagus nerve-splenic axis, glial circuits in the dorsal root ganglia, and autonomic inflammatory loops, opens a new avenue for managing postsurgical inflammation and nociceptive potentiation [160,162]. Advances in biomaterial science, electrical signal decoding, and design of minimally invasive leads are expected to support selective modulation of molecular targets without reliance on systemic drugs.

### 9.3. Regenerative Neuroimmune Approaches

The integration of regenerative medicine and neuromodulation represents a new therapeutic approach aimed at repairing dysfunctional pain circuits. Mesenchymal stem cell-derived exosomes exhibit strong neuroregenerative and anti-inflammatory properties, including the inhibition of microglial activation and the regulation of synaptic homeostasis and axonal regeneration [163,164]. Gene-targeted therapies that focus on modulating ion channel genes, neurotransmitters, and glial genes may be tested for use with activity-dependent expression systems and neuromodulation [165,166,167]. These hybrid approaches may enable long-lasting modification of maladaptive nociceptive circuits and offer potential solutions for refractory neuropathic pain and chronic postsurgical pain syndromes.

### 9.4. Personalized Neural Signatures and AI-Based Analgesia Titration

Advances in personalized neuromodulation will rely on measurable brain and neural signals to predict clinical response to intervention. Machine learning algorithms have already demonstrated the ability to classify pain states based on quantitative sensory testing parameters, conduction properties, evoked response properties, and cortical oscillations [168,169,170]. Future developments may involve the use of these biomarkers to guide parameter selection for specific therapies, such as in peripheral nerve stimulation, focused ultrasound stimulation, photobiomodulation therapy, and transcranial stimulation. Additionally, machine learning algorithms can adjust stimulation parameters to maintain therapeutic effects while minimizing energy use, fatigue, and potential overstimulation of target tissues [170,171,172].

### 9.5. Hybrid Pharmacologic–Neuromodulation Strategies

The combination of nonpharmacologic approaches and specific pharmaceuticals for synergistic analgesia is promising. Neuromodulation may reduce the dose requirements for ketamine, local anesthetics, and anti-inflammatory medications for effective management of postoperative pain [46,50]. Pharmaceuticals may potentiate the molecular effects of heat-related modalities such as radiofrequency ablation and ultrasound energy [173]. Combinations such as low-dose ketamine with focused ultrasound may enhance suppression of early central sensitization. Photobiomodulation may enhance the anti-inflammatory properties of conventional nonsteroidal analgesics, facilitating recovery of musculoskeletal function after orthopedic surgery [120,173,174]. Cryoneurolysis combined with peripheral nerve stimulation and perineural corticosteroids may be more effective in sustaining the benefits of opioid-free analgesia in major surgery protocols [46,50]. Collectively, these approaches represent an evolving paradigm for more effective multimodal analgesia.

### 9.6. Physiologic Guidance of Perioperative Analgesia and Nociception Monitoring

Physiologic monitoring of nociception and antinociception has emerged as an important strategy for optimizing perioperative pain management. Tools such as the Surgical Pleth Index, pupillometry, the Nociception Level index, and the Analgesia Nociception Index have been investigated to guide intraoperative analgesic titration through real-time assessment of autonomic responses to surgical stimuli [156,157,175,176,177]. Among these modalities, the Surgical Pleth Index has the most widespread use in clinical anesthesia practice due to ease of integration into standard anesthetic monitoring methods [175]. By reducing periods of nociceptive under- or over-treatment, nociception-guided anesthesia may help limit central sensitization and maladaptive pain processing that contribute to chronic postsurgical pain, although definitive outcome data remain limited [3,159]. These monitoring approaches represent a complementary, nonpharmacologic strategy that aligns with precision analgesia and established multimodal perioperative pain management.

## 10. Conclusions

Nonpharmacologic analgesic technologies offer mechanism-based, opioid-sparing adjuncts for perioperative and chronic pain management. By engaging peripheral, spinal, supraspinal, and neuroimmune processes that are not directly targeted by systemic pharmacotherapy, these approaches broaden the scope of anesthesia-based pain care. Human evidence supporting these modalities is heterogeneous, ranging from perioperative randomized trials for select interventions to postoperative, chronic pain, and experimental studies for others. Clinical integration will be guided by procedure-specific evidence, patient selection, and alignment with established multimodal analgesia frameworks. Future progress will depend on standardized protocols, mechanistic validation, cost considerations, and improved methods for identifying patients most likely to benefit. Ongoing advances in closed-loop systems, bioelectronic medicine, and adaptive neuromodulation suggest a transition toward precision, physiology-driven analgesia. Together, these developments establish nonpharmacologic technologies as integral components of modern, mechanism-based pain management strategies.

## Figures and Tables

**Table 1:** Comparison of nonpharmacologic analgesic technologies used in perioperative and chronic pain management.

Modality	Primary Target Level	Mechanism of Action	Typical Clinical Indications	Invasiveness	Typical Dosing/Parameters (High-Level)	Human Perioperative Evidence (Study Type)	Key Risks/Contraindications	Perioperative Use-Case
Peripheral Nerve Stimulation (PNS)	Peripheral nerve, DRG	Electrical modulation of Aβ afferents; suppression of ectopic firing; neuroimmune modulation	Postsurgical neuropathic pain, postamputation pain, focal neuralgia	Minimally invasive (percutaneous)	Hz-range stimulation; amplitude titrated to sensory threshold; days–weeks	Moderate; human perioperative evidence limited to pilot studies and registered clinical trials (perioperative trials referenced in Section 8.5; NCT03481725, NCT03484429)	Infection, lead migration, bleeding, device interference	Pre-op or post-op opioid-sparing analgesia; CPSP prevention
TENS/Interferential Stimulation	Peripheral afferents, dorsal horn	Sensory gating; endogenous opioid release; enhanced descending inhibition	Acute postoperative pain, musculoskeletal pain	Noninvasive	Surface electrodes; daily sessions; low–moderate intensity	Moderate; supported by human perioperative randomized trials summarized in a systematic review and meta-analysis [16] and prior perioperative critical reviews [48] demonstrating reduced postoperative pain and opioid consumption	Skin irritation; limited efficacy in deep pain	PACU and early recovery adjunct
tDCS/tACS/TMS	Cortical and supraspinal networks	Modulation of cortical excitability and oscillatory activity; enhanced descending inhibition	Central sensitization, chronic pain, perioperative modulation	Noninvasive	Session-based stimulation over motor/prefrontal cortex	Emerging; supported by human experimental and chronic pain clinical studies [30,32,49]	Seizure risk (rare), headache; implanted devices	Adjunct for central pain modulation, CPSP risk
Cryoneurolysis	Peripheral nerve	Reversible axonal conduction block; reduced neurogenic inflammation	TKA, thoracic surgery, rib fractures	Minimally invasive	Single application; −60 °C to −100 °C; weeks–months effect	Moderate; supported by human perioperative randomized controlled trials and registry studies, including mastectomy [50], rib fracture analgesia [51], and total knee arthroplasty [52]	Sensory loss, neuropraxia, cold injury	Pre-op long-duration analgesia
Pulsed/Continuous Radiofrequency (PRF/CRF)	Peripheral nerve, medial branches	PRF: neuromodulation without ablation; CRF: thermal neurodestruction	Facet pain, neuropathic pain	Minimally invasive	PRF < 42 °C; CRF 60–90 °C	Moderate; supported by human chronic and subacute interventional pain studies [53,54,55], with limited perioperative application [56]	Neural injury, neuritis, thermal damage	Selective perioperative or chronic pain adjunct
Photobiomodulation (PBM)	Peripheral tissue, mitochondria, glia	Mitochondrial activation; reduced oxidative stress and cytokine signaling	Postoperative pain, soft tissue injury, neuropathic pain	Noninvasive	Wavelength-specific LED/laser; repeated sessions	Emerging; human clinical evidence includes peri-procedural and postoperative studies with heterogeneous designs [57], while high-quality perioperative surgical randomized trials remain limited	Eye injury (if misused); minimal systemic risk	Post-op recovery and rehabilitation
Low-Intensity Focused Ultrasound (LIFU)	Deep cortical and subcortical targets	Mechanosensitive ion channel modulation; neuroimmune effects	Central pain modulation, experimental perioperative use	Noninvasive	Image-guided acoustic pulses; parameter-sensitive	Experimental; human studies cited are limited to non-surgical and investigational neuromodulation contexts, with no established perioperative randomized surgical trials	Off-target neuromodulation, imaging needs	Future intra-op or closed-loop systems
Extracorporeal Shockwave Therapy (ESWT)	Musculoskeletal and peripheral tissues	Mechanical disruption; angiogenesis; reduced nociceptor firing	Tendinopathy, musculoskeletal pain	Noninvasive	Session-based acoustic pulses	Moderate; supported by human clinical studies demonstrating postoperative musculoskeletal rehabilitation and pain reduction [58,59,60], with limited direct evidence for acute perioperative analgesia	Local pain, hematoma	Post-op rehabilitation adjunct
Pulsed Electromagnetic Field Therapy (PEMF)	Cellular membranes, immune cells	Modulation of transmembrane potentials; anti-inflammatory effects	Postsurgical pain, wound healing	Noninvasive	Repeated low-intensity field exposure	Emerging; limited human perioperative randomized data cited, including surgical populations [61], primarily supporting postoperative recovery and wound healing	Minimal; device interference	Adjunct in recovery and wound healing

## Data Availability

No new data were generated or analyzed in this study.

## References

[1] Chou R., Gordon D.B., de Leon-Casasola O.A., Rosenberg J.M., Bickle S., Brennan T., Carter T., Cassidy C.L., Chittenden E.H., Degenhardt E. (2016). Management of Postoperative Pain: A Clinical Practice Guideline From the American Pain Society, the American Society of Regional Anesthesia and Pain Medicine, and the American Society of Anesthesiologists’ Committee on Regional Anesthesia, Executive Committee, and Administrative Council. J. Pain.

[2] Pirie K., Traer E., Finniss D., Myles P.S., Riedel B. (2022). Current approaches to acute postoperative pain management after major abdominal surgery: A narrative review and future directions. Br. J. Anesth..

[3] Tassou A., Richebe P., Rivat C. (2025). Mechanisms of chronic postsurgical pain. Reg. Anesth. Pain Med..

[4] Silva J.R., Iftinca M., Gomes F.I.F., Segal J.P., Smith O.M.A., Bannerman C.A., Mendes A.S., Defaye M., Robinson M.E.C., Gilron I. (2022). Skin-resident dendritic cells mediate postoperative pain via CCR4 on sensory neurons. Proc. Natl. Acad. Sci. USA.

[5] Echeverria-Villalobos M., Tortorici V., Brito B.E., Ryskamp D., Uribe A., Weaver T. (2023). The role of neuroinflammation in the transition of acute to chronic pain and the opioid-induced hyperalgesia and tolerance. Front. Pharmacol..

[6] Colvin L.A., Bull F., Hales T.G. (2019). Perioperative opioid analgesia-when is enough too much? A review of opioid-induced tolerance and hyperalgesia. Lancet.

[7] Mercadante S., Arcuri E., Santoni A. (2019). Opioid-Induced Tolerance and Hyperalgesia. CNS Drugs.

[8] Adams T.J., Aljohani D.M., Forget P. (2023). Perioperative opioids: A narrative review contextualising new avenues to improve prescribing. Br. J. Anesth..

[9] Zhang Z., Zhanyu N., Dong S. (2025). Spinal glia-driven neuroinflammation as a therapeutic target for neuropathic pain: Rational development of novel analgesics. Neurosci. Biobehav. Rev..

[10] Calvo M., Dawes J.M., Bennett D.L.H. (2012). The role of the immune system in the generation of neuropathic pain. Lancet Neurol..

[11] Jain A., Hakim S., Woolf C.J. (2024). Immune drivers of physiological and pathological pain. J. Exp. Med..

[12] Alessi I., Banton K.L., J S., C Z.-M., Ch P., Rj R., D B.-O. (2025). Exploring novel non-opioid pathways and therapeutics for pain modulation. Mol. Pain.

[13] Amaya F., Izumi Y., Matsuda M., Sasaki M. (2013). Tissue injury and related mediators of pain exacerbation. Curr. Neuropharmacol..

[14] Petho G., Reeh P.W. (2012). Sensory and signaling mechanisms of bradykinin, eicosanoids, platelet-activating factor, and nitric oxide in peripheral nociceptors. Physiol. Rev..

[15] Chen S.-H., Lin Y.-W., Tseng W.-L., Lin W.-T., Lin S.-C., Hsueh Y.-Y. (2024). Ultrahigh frequency transcutaneous electrical nerve stimulation for neuropathic pain alleviation and neuromodulation. Neurotherapeutics.

[16] Smith T.J., Wang E.J., Loprinzi C.L. (2023). Cutaneous Electroanalgesia for Relief of Chronic and Neuropathic Pain. N. Engl. J. Med..

[17] Cathenaut L., Schlichter R., Hugel S. (2023). Short-term plasticity in the spinal nociceptive system. Pain.

[18] Kuner R. (2015). Spinal excitatory mechanisms of pathological pain. Pain.

[19] Cho J.H., Lee D.G. (2020). Translocation of AMPA Receptors in the Dorsal Horn of the Spinal Cord Corresponding to Long-term Depression Following Pulsed Radiofrequency Stimulation at the Dorsal Root Ganglion. Pain Med..

[20] Jensen T.S., Finnerup N.B. (2014). Allodynia and hyperalgesia in neuropathic pain: Clinical manifestations and mechanisms. Lancet Neurol..

[21] Knotkova H., Hamani C., Sivanesan E., Le Beuffe M.F.E., Moon J.Y., Cohen S.P., Huntoon M.A. (2021). Neuromodulation for chronic pain. Lancet.

[22] Vallejo R., Bradley K., Kapural L. (2017). Spinal Cord Stimulation in Chronic Pain: Mode of Action. Spine.

[23] Gosselin R.-D., Suter M.R., Ji R.-R., Decosterd I. (2010). Glial cells and chronic pain. Neuroscientist.

[24] Ji R.-R., Berta T., Nedergaard M. (2013). Glia and pain: Is chronic pain a gliopathy?. Pain.

[25] Donnelly C.R., Andriessen A.S., Chen G., Wang K., Jiang C., Maixner W., Ji R.-R. (2020). Central Nervous System Targets: Glial Cell Mechanisms in Chronic Pain. Neurotherapeutics.

[26] Hanani M., Spray D.C. (2020). Emerging importance of satellite glia in nervous system function and dysfunction. Nat. Rev. Neurosci..

[27] Ketz A.K., Byrnes K.R., Grunberg N.E., Kasper C.E., Osborne L., Pryor B., Tosini N.L., Wu X., Anders J.J. (2017). Characterization of Macrophage/Microglial Activation and Effect of Photobiomodulation in the Spared Nerve Injury Model of Neuropathic Pain. Pain Med..

[28] Malta I., Moraes T., Elisei L., Novaes R., Galdino G. (2022). Investigation of the effects of therapeutic ultrasound or photobiomodulation and the role of spinal glial cells in osteoarthritis-induced nociception in mice. Lasers Med. Sci..

[29] Tao X., Lee M.S., Donnelly C.R., Ji R.-R. (2020). Neuromodulation, Specialized Proresolving Mediators, and Resolution of Pain. Neurotherapeutics.

[30] Knotkova H., Nitsche M.A., Cruciani R.A. (2013). Putative physiological mechanisms underlying tDCS analgesic effects. Front. Hum. Neurosci..

[31] Takeuchi N. (2023). Pain control based on oscillatory brain activity using transcranial alternating current stimulation: An integrative review. Front. Hum. Neurosci..

[32] Mylius V., Borckardt J.J., Lefaucheur J.-P. (2012). Noninvasive cortical modulation of experimental pain. Pain.

[33] Zhang T., Wang Z., Liang H., Wu Z., Li J., Ou-Yang J., Yang X., Peng Y.B., Zhu B. (2022). Transcranial Focused Ultrasound Stimulation of Periaqueductal Gray for Analgesia. IEEE Trans. Biomed. Eng..

[34] Zeng X., Powell R., Woolf C.J. (2025). Mechanism-based nonopioid analgesic targets. J. Clin. Investig..

[35] Shi Y., Wu W. (2023). Multimodal non-invasive non-pharmacological therapies for chronic pain: Mechanisms and progress. BMC Med..

[36] Peng W.W., Tang Z.Y., Zhang F.R., Li H., Kong Y.Z., Lannetti G.D., Hu L. (2019). Neurobiological mechanisms of TENS-induced analgesia. Neuroimage.

[37] Cardoso F.D.S., Salehpour F., Coimbra N.C., Gonzalez-Lima F., da Silva S.G. (2022). Photobiomodulation for the treatment of neuroinflammation: A systematic review of controlled laboratory animal studies. Front. Neurosci..

[38] Fiore N.T., Debs S.R., Hayes J.P., Duffy S.S., Moalem-Taylor G. (2023). Pain-resolving immune mechanisms in neuropathic pain. Nat. Rev. Neurol..

[39] Mika J., Zychowska M., Popiolek-Barczyk K., Rojewska E., Przewlocka B. (2013). Importance of glial activation in neuropathic pain. Eur. J. Pharmacol..

[40] Vanderwall A.G., Milligan E.D. (2019). Cytokines in Pain: Harnessing Endogenous Anti-Inflammatory Signaling for Improved Pain Management. Front. Immunol..

[41] Lyu Z., Guo Y., Gong Y., Fan W., Duo B., Li N., Wang S., Xu Y., Liu Y., Chen B. (2021). The Role of Neuroglial Crosstalk and Synaptic Plasticity-Mediated Central Sensitization in Acupuncture Analgesia. Neural Plast..

[42] Fitzcharles M.-A., Cohen S.P., Clauw D.J., Littlejohn G., Usui C., Hauser W. (2021). Nociplastic pain: Towards an understanding of prevalent pain conditions. Lancet.

[43] Flood A., Cavaleri R., Chang W.-J., Kutch J., Toufexis C., Summers S.J. (2025). Noninvasive brain stimulation beyond the motor cortex: A systematic review and meta-analysis exploring effects on quantitative sensory testing in clinical pain. Pain Med..

[44] Finnerup N.B. (2019). Nonnarcotic Methods of Pain Management. N. Engl. J. Med..

[45] Edwards D.A., Hedrick T.L., Jayaram J., Argoff C., Gulur P., Holubar S.D., Gan T.J., Mythen M.G., Miller T.E., Shaw A.D. (2019). American Society for Enhanced Recovery and Perioperative Quality Initiative Joint Consensus Statement on Perioperative Management of Patients on Preoperative Opioid Therapy. Anesth. Analg..

[46] Joshi G.P. (2023). Rational Multimodal Analgesia for Perioperative Pain Management. Curr. Pain Headache Rep..

[47] Gabriel R.A., Swisher M.W., Sztain J.F., Furnish T.J., Ilfeld B.M., Said E.T. (2019). State of the art opioid-sparing strategies for post-operative pain in adult surgical patients. Expert Opin. Pharmacother..

[48] Liu S., Long S.-S., Li F., Yang H., Pu S., Du D., Luo X., Zhang Y.-Q., Han Q. (2025). Neural basis of transcutaneous electrical nerve stimulation for neuropathic pain relief. Neuron.

[49] Xiong H.-Y., Zheng J.-J., Wang X.-Q. (2022). Non-invasive Brain Stimulation for Chronic Pain: State of the Art and Future Directions. Front. Mol. Neurosci..

[50] Finneran J.J., Ilfeld B.M. (2025). Role of peripheral nerve stimulation and percutaneous cryoneurolysis in preventing chronic postsurgical pain. Reg. Anesth. Pain Med..

[51] Finneran J.J., Kobayashi L., Costantini T.W., Weaver J.L., Berndtson A.E., Haines L., Doucet J.J., Adams L., Santorelli J.E., Lee J. (2025). Ultrasound-guided Percutaneous Cryoneurolysis for the Treatment of Pain after Traumatic Rib Fracture: A Randomized, Active-controlled, Participant- and Observer-masked Study. Anesthesiology.

[52] Moon J.T., Li H., Cooper M., Huang J., Prologo J.D. (2025). Cryoneurolysis as a Neuroregenerative Intervention for Chronic Painful Mononeuropathies: A Four-Patient Case Series and Discussion. Cardiovasc. Interv. Radiol..

[53] Sam J., Catapano M., Sahni S., Ma F., Abd-Elsayed A., Visnjevac O. (2021). Pulsed Radiofrequency in Interventional Pain Management: Cellular and Molecular Mechanisms of Action—An Update and Review. Pain Physician.

[54] Huang R.-Y., Liao C.-C., Tsai S.-Y., Yen C.-T., Lin C.-W., Chen T.-C., Lin W.-T., Chang C.-H., Wen Y.-R. (2017). Rapid and Delayed Effects of Pulsed Radiofrequency on Neuropathic Pain: Electrophysiological, Molecular, and Behavioral Evidence Supporting Long-Term Depression. Pain Physician.

[55] Jiang R., Li P., Yao Y.-X., Li H., Liu R., Huang L.-E., Ling S., Peng Z., Yang J., Zha L. (2019). Pulsed radiofrequency to the dorsal root ganglion or the sciatic nerve reduces neuropathic pain behavior, decreases peripheral pro-inflammatory cytokines and spinal β-catenin in chronic constriction injury rats. Reg. Anesth. Pain Med..

[56] Lee D.W., Pritzlaff S., Jung M.J., Ghosh P., Hagedorn J.M., Tate J., Scarfo K., Strand N., Chakravarthy K., Sayed D. (2021). Latest Evidence-Based Application for Radiofrequency Neurotomy (LEARN): Best Practice Guidelines from the American Society of Pain and Neuroscience (ASPN). J. Pain Res..

[57] Zachar J.J., Reher P., Zafar S., Walsh L.J. (2025). Challenges in evaluating the analgesic effects of photobiomodulation in dentistry: A narrative review. J. Dent..

[58] d’Agostino M.C., Craig K., Tibalt E., Respizzi S. (2015). Shock wave as biological therapeutic tool: From mechanical stimulation to recovery and healing, through mechanotransduction. Int. J. Surg..

[59] Charles R., Fang L., Zhu R., Wang J. (2023). The effectiveness of shockwave therapy on patellar tendinopathy, Achilles tendinopathy, and plantar fasciitis: A systematic review and meta-analysis. Front. Immunol..

[60] Speed C. (2014). A systematic review of shockwave therapies in soft tissue conditions: Focusing on the evidence. Br. J. Sports Med..

[61] Rohde C.H., Taylor E.M., Alonso A., Ascherman J.A., Hardy K.L., Pilla A.A. (2015). Pulsed electromagnetic fields reduce postoperative interleukin-1β, pain, and inflammation: A double-blind, placebo-controlled study in TRAM flap breast reconstruction patients. Plast. Reconstr. Surg..

[62] Sio L.C.O., Hom B., Garg S., Abd-Elsayed A. (2023). Mechanism of Action of Peripheral Nerve Stimulation for Chronic Pain: A Narrative Review. Int. J. Mol. Sci..

[63] Lin T., Gargya A., Singh H., Sivanesan E., Gulati A. (2020). Mechanism of Peripheral Nerve Stimulation in Chronic Pain. Pain Med..

[64] Karcz M., Gharibo C. (2024). Peripheral Nervous System Pain Modulation. Curr. Neuropharmacol..

[65] Ilfeld B.M., Finneran J.J. (2020). Cryoneurolysis and Percutaneous Peripheral Nerve Stimulation to Treat Acute Pain. Anesthesiology.

[66] Kaye A.D., Plaisance T.R., Smith S.A., Ragland A.R., Alfred M.J., Nguyen C.G., Chami A.A., Kataria S., Dufrene K., Shekoohi S. (2024). Peripheral Nerve Stimulation in Postoperative Analgesia: A Narrative Review. Curr. Pain Headache Rep..

[67] Abd-Elsayed A., Attanti S., Anderson M., Dunn T., Maloney J., Strand N. (2024). Mechanism of Action of Temporary Peripheral Nerve Stimulation. Curr. Pain Headache Rep..

[68] Wong C.-E., Hu C.-Y., Lee P.-H., Huang C.-C., Huang H.-W., Huang C.-Y., Lo H.-T., Liu W., Lee J.-S. (2022). Sciatic nerve stimulation alleviates acute neuropathic pain via modulation of neuroinflammation and descending pain inhibition in a rodent model. J. Neuroinflammation.

[69] Wong C.-E., Liu W., Huang C.-C., Lee P.-H., Huang H.-W., Chang Y., Lo H.-T., Chen H.-F., Kuo L.-C., Lee J.-S. (2024). Sciatic nerve stimulation alleviates neuropathic pain and associated neuroinflammation in the dorsal root ganglia in a rodent model. J. Transl. Med..

[70] Chakravarthy K.V., Xing F., Bruno K., Kent A.R., Raza A., Hurlemann R., Knife T.M. (2019). A Review of Spinal and Peripheral Neuromodulation and Neuroinflammation: Lessons Learned Thus Far and Future Prospects of Biotype Development. Neuromodulation.

[71] Yang Q.-H., Zhang Y.-H., Du S.-H., Wang Y.-C., Fang Y., Wang X.-Q. (2022). Non-invasive Brain Stimulation for Central Neuropathic Pain. Front. Mol. Neurosci..

[72] Strohman A., Legon W. (2025). Neuromodulation of the Cingulate Cortex for Pain. Neuroscientist.

[73] Lim Y.-S., Kim J.-H., Kim J., Hoang M., Kang W., Koh M., Choi W.H., Park S., Jeong U., Kim D.H. (2025). Precise control of tibial nerve stimulation for bladder regulation via evoked compound action potential feedback mechanisms. Nat. Commun..

[74] Montenegro T.S., Ali R., Arle J.E. (2022). Closed-Loop Systems in Neuromodulation: Electrophysiology and Wearables. Neurosurg. Clin. N. Am..

[75] Zanos S. (2019). Closed-Loop Neuromodulation in Physiological and Translational Research. Cold Spring Harb. Perspect. Med..

[76] Beauchene C., Zurn C.A., Ehrens D., Duff I., Duan W., Caterina M., Guan Y., Sarma S.V. (2023). Steering Toward Normative Wide-Dynamic-Range Neuron Activity in Nerve-Injured Rats with Closed-Loop Peripheral Nerve Stimulation. Neuromodulation.

[77] Chakravarthy K., Ritcher H., Christo P.J., Williams K., Guan Y. (2018). Spinal Cord Stimulation for Treating Chronic Pain: Reviewing Preclinical and Clinical Data on Paresthesia-Free High-Frequency Therapy. Neuromodulation.

[78] Kapural L., Yu C., Doust M.W., Gliner B.E., Vallejo R., Sitzman B.T., Amirdelfan K., Morgan D.M., Brown L.L., Yearwood T.L. (2015). Novel 10-kHz High-frequency Therapy (HF10 Therapy) Is Superior to Traditional Low-frequency Spinal Cord Stimulation for the Treatment of Chronic Back and Leg Pain: The SENZA-RCT Randomized Controlled Trial. Anesthesiology.

[79] Lee K.Y., Bae C., Lee D., Kagan Z., Bradley K., Chung J.M., La J.-H. (2020). Low-intensity, Kilohertz Frequency Spinal Cord Stimulation Differently Affects Excitatory and Inhibitory Neurons in the Rodent Superficial Dorsal Horn. Neuroscience.

[80] Yu J., Wong S., Lin Z., Shan Z., Fan C., Xia Z., Cheung M., Zhu X., Liu J.A., Cheung C.W. (2024). High-Frequency Spinal Stimulation Suppresses Microglial Kaiso-P2X7 Receptor Axis-Induced Inflammation to Alleviate Neuropathic Pain in Rats. Ann. Neurol..

[81] Peeters J.-B., Raftopoulos C. (2020). Tonic, Burst, High-Density, and 10-kHz High-Frequency Spinal Cord Stimulation: Efficiency and Patients’ Preferences in a Failed Back Surgery Syndrome Predominant Population. Review of Literature. World Neurosurg..

[82] Mekhail N., Levy R.M., Deer T.R., Kapural L., Li S., Amirdelfan K., Hunter C.W., Rosen S.M., Costandi S.J., Falowski S.M. (2022). Durability of Clinical and Quality-of-Life Outcomes of Closed-Loop Spinal Cord Stimulation for Chronic Back and Leg Pain: A Secondary Analysis of the Evoke Randomized Clinical Trial. JAMA Neurol..

[83] DeSantana J.M., Da Silva L.F.S., De Resende M.A., Sluka K.A. (2009). Transcutaneous electrical nerve stimulation at both high and low frequencies activates ventrolateral periaqueductal grey to decrease mechanical hyperalgesia in arthritic rats. Neuroscience.

[84] De Oliveira M.E., Da Silva J.T., Brioschi M.L., Chacur M. (2021). Effects of photobiomodulation therapy on neuropathic pain in rats: Evaluation of nociceptive mediators and infrared thermography. Lasers Med. Sci..

[85] Fishman M.A., Scherer A.M., Katsarakes A.M., Larson L., Kim P.S. (2021). Temperature-Mediated Nerve Blocks in the Treatment of Pain. Curr. Pain Headache Rep..

[86] Cheng K., Martin L.F., Slepian M.J., Patwardhan A.M., Ibrahim M.M. (2021). Mechanisms and Pathways of Pain Photobiomodulation: A Narrative Review. J. Pain.

[87] Buzza A.S., Cousins H., Tapas K.E., Anders J.J., Lewis S.J., Jenkins M.W., Moffitt M.A. (2024). Direct Photobiomodulation Therapy on the Sciatic Nerve Significantly Attenuates Acute Nociceptive Sensitivity Without Affecting Motor Output. Neuromodulation.

[88] Garibyan L., Tuchayi S.M., Wang Y., Khodorova A., Stemmer-Rachamimov A., Purschke M., Osseiran S., Evans C.L., Mao J., Strichartz G. (2020). Neural Selective Cryoneurolysis with Ice Slurry Injection in a Rat Model. Anesthesiology.

[89] Hsu M., Stevenson F.F. (2015). Wallerian degeneration and recovery of motor nerves after multiple focused cold therapies. Muscle Nerve.

[90] Zhang Z., Lyon T.D., Kadow B.T., Shen B., Wang J., Lee A., Kang A., Roppolo J.R., de Groat W.C., Tai C. (2016). Conduction block of mammalian myelinated nerve by local cooling to 15–30 °C after a brief heating. J. Neurophysiol..

[91] Ng M.K., Lin J.H., Spitzer A.I., Dasa V., Rivadeneyra A., Rogenmoser D., Concoff A.L., DiGiorgi M., Urban J., Mihalko W.M. (2025). Preoperative Cryoneurolysis Improves Pain and Function for at Least 12 Months after Total Knee Arthroplasty: A Multicenter Registry Study. J. Arthroplast..

[92] Hajiaghajani S., Poursalehian M., Samakoosh A.N., Bahrami O., Hecht C.J., Kamath A.F. (2025). Does Preoperative Anterior Genicular Nerve Cryoneurolysis Improve Early Outcomes of Primary Total Knee Arthroplasty? A Systematic Review and Meta-Analysis. J. Arthroplast..

[93] Sayed D., Grider J., Strand N., Hagedorn J.M., Falowski S., Lam C.M., Francio V.T., Beall D.P., Tomycz N.D., Davanzo J.R. (2022). The American Society of Pain and Neuroscience (ASPN) Evidence-Based Clinical Guideline of Interventional Treatments for Low Back Pain. J. Pain Res..

[94] de Freitas L.F., Hamblin M.R. (2016). Proposed mechanisms of photobiomodulation or low-level light therapy. IEEE J. Sel. Top. Quantum Electron..

[95] Hamblin M.R. (2018). Mechanisms and mitochondrial redox signaling in photobiomodulation. Photochem. Photobiol..

[96] Maghfour J., Ozog D.M., Mineroff J., Jagdeo J., Kohli I., Lim H.W. (2024). Photobiomodulation CME part I: Overview and mechanism of action. J. Am. Acad. Dermatol..

[97] Andreo L., Soldera C.B., Ribeiro B.G., de Matos P.R.V., Bussadori S.K., Fernandes K.P.S., Mesquita-Ferrari R.A. (2017). Effects of photobiomodulation on experimental models of peripheral nerve injury. Lasers Med. Sci..

[98] Lacerda-Santos J.T., Granja G.L., Firmino R.T., Dias R.F., Melo D.P., Granville-Garcia A.F., Martins C.C. (2023). Use of photobiomodulation to reduce postoperative pain, edema, and trismus after third molar surgery: A systematic review and meta-analysis. J. Oral Maxillofac. Surg..

[99] De Oliveira M.F., Johnson D.S., Demchak T., Tomazoni S.S., Leal-Junior E.C. (2022). Low-intensity laser and LED (photobiomodulation therapy) for pain control of the most common musculoskeletal conditions. Eur. J. Phys. Rehabil. Med..

[100] Hsieh Y.-L., Chou L.-W., Chang P.-L., Yang C.-C., Kao M.-J., Hong C.-Z. (2012). Low-level laser therapy alleviates neuropathic pain and promotes function recovery in rats with chronic constriction injury: Possible involvements in hypoxia-inducible factor 1α (HIF-1α). J. Comp. Neurol..

[101] Buntragulpoontawee M., Chang K.-V., Vitoonpong T., Pornjaksawan S., Kitisak K., Saokaew S., Kanchanasurakit S. (2021). The effectiveness and safety of commonly used injectates for ultrasound-guided hydrodissection treatment of peripheral nerve entrapment syndromes: A systematic review. Front. Pharmacol..

[102] Colorado B., McNeill D., Norbury J. (2025). Ultrasound-guided nerve hydrodissection for peripheral entrapment neuropathies. Muscle Nerve.

[103] Lam S.K.H., Reeves K.D., Cheng A.-L. (2017). Transition from deep regional blocks toward deep nerve hydrodissection in the upper body and torso: Method description and results from a retrospective chart review of the analgesic effect of 5% dextrose water as the primary hydrodissection injectate to enhance safety. BioMed Res. Int..

[104] Dufour E., Donat N., Jaziri S., Kurdi O., Couturier C., Dreyfus J.-F., Fischler M. (2012). Ultrasound-guided perineural circumferential median nerve block with and without prior dextrose 5% hydrodissection: A prospective randomized double-blinded noninferiority trial. Anesth. Analg..

[105] Shi Y., Wu W. (2025). Advances and prospects of transcranial focused ultrasound in pain neuromodulation. Pain.

[106] Mattoso-Camara A.R., Papini J.Z.B., Teixeira M.A., Fujii D.N., Tofoli G.R., Garcez A.S. (2025). Targeting pain and inflammation: A comparative study of photobiomodulation with 532 and 660 nm lasers in rats. Photochem. Photobiol.

[107] Ramezani F., Neshastheh-Riz A., Ghadaksaz A., Moghadas Fazeli S., Janzadeh A., Hamblin M.R. (2022). Mechanistic aspects of photobiomodulation therapy in the nervous system. Lasers Med. Sci..

[108] Dell’Italia J., Sanguinetti J.L., Monti M.M., Bystritsky A., Reggente N. (2022). Current state of potential mechanisms supporting low-intensity focused ultrasound for neuromodulation. Front. Hum. Neurosci..

[109] Yoo S., Mittelstein D.R., Hurt R.C., Lacroix J., Shapiro M.G. (2022). Focused ultrasound excites cortical neurons via mechanosensitive calcium accumulation and ion channel amplification. Nat. Commun..

[110] Newman M., Rasiah P.K., Kusunose J., Rex T.S., Mahadevan-Jansen A., Hardenburger J., Jansen E.D., Millis B., Caskey C.F. (2024). Ultrasound modulates calcium activity in cultured neurons, glial cells, endothelial cells and pericytes. Ultrasound Med. Biol..

[111] Bao J., Byraju K., Patel V.J., Hellman A., Neubauer P., Burdette C., Rafferty E., Park Y.L., Trowbridge R., Shin D.S. (2022). The effects of low-intensity focused ultrasound on neuronal activity in pain processing regions in a rodent model of common peroneal nerve injury. Neurosci. Lett..

[112] Hellman A., Clum A., Maietta T., Srikanthan A., Patel V., Panse D., Zimmerman O., Neubauer P., Nalwalk J., Williams E. (2021). Effects of external low-intensity focused ultrasound on inflammatory markers in neuropathic pain. Neurosci. Lett..

[113] Phan T.T., Shin S., Kim H.J., Lee K., Kim T.-Y., Lee J.-H., Kang D.-W., Shin H., Lee H.E., Jayathilake N.J. (2025). Harnessing theta–gamma coupled brainwaves using ultrasound for spinal astrocyte revitalization and sustained neuropathic pain relief in mice. Nat. Commun..

[114] Mishra A., Yang P.-F., Manuel T.J., Newton A.T., Phipps M.A., Luo H., Sigona M.K., Reed J.L., Gore J.C., Grissom W.A. (2023). Disrupting nociceptive information processing flow through transcranial focused ultrasound neuromodulation of thalamic nuclei. Brain Stimul..

[115] In A., Strohman A., Payne B., Legon W. (2024). Low-intensity focused ultrasound to the posterior insula reduces temporal summation of pain. Brain Stimul..

[116] Xu H.-R., Yi Y.-L., Xue C., Guo Z.-Q., Ding L., Jia J. (2025). The efficacy and mechanisms of low-intensity transcranial ultrasound stimulation on pain: A systematic review of human and animal studies. J. Headache Pain.

[117] Cox S.S., Connolly D.J., Peng X., Badran B.W. (2025). A comprehensive review of low-intensity focused ultrasound parameters and applications in neurologic and psychiatric disorders. Neuromodulation.

[118] Osada T., Konishi S. (2024). Noninvasive intervention by transcranial ultrasound stimulation: Modulation of neural circuits and its clinical perspectives. Psychiatry Clin. Neurosci..

[119] Cooper L., Gil Malinao M., Hong G. (2024). Force-based neuromodulation. Acc. Chem. Res..

[120] Todd N., McDannold N., Borsook D. (2020). Targeted manipulation of pain neural networks: The potential of focused ultrasound for treatment of chronic pain. Neurosci. Biobehav. Rev..

[121] Mariotto S., Carcereri de Prati A., Cavalieri E., Amelio E., Marlinghaus E., Suzuki H. (2009). Extracorporeal shock wave therapy in inflammatory diseases: Molecular mechanism that triggers anti-inflammatory action. Curr. Med. Chem..

[122] Medina C. (2023). Shockwave therapy in veterinary rehabilitation. Vet. Clin. N. Am. Small Anim. Pract..

[123] Brown M.R.D., Farquhar-Smith P., Williams J.E., ter Haar G., deSouza N.M. (2015). The use of high-intensity focused ultrasound as a novel treatment for painful conditions: A description and narrative review of the literature. Br. J. Anesth..

[124] Huisman M., Staruch R.M., Ladouceur-Wodzak M., van den Bosch M.A., Burns D.K., Chhabra A., Chopra R. (2015). Non-invasive targeted peripheral nerve ablation using 3D MR neurography and MRI-guided high-intensity focused ultrasound (MR-HIFU): Pilot study in a swine model. PLoS ONE.

[125] Ross C.L., Teli T., Harrison B.S. (2016). Electromagnetic field devices and their effects on nociception and peripheral inflammatory pain mechanisms. Altern. Ther. Health Med..

[126] Costantini E., Sinjari B., D’Angelo C., Murmura G., Reale M., Caputi S. (2019). Human gingival fibroblasts exposed to extremely low-frequency electromagnetic fields: In vitro model of wound-healing improvement. Int. J. Mol. Sci..

[127] Caliogna L., Medetti M., Bina V., Brancato A.M., Castelli A., Jannelli E., Ivone A., Gastaldi G., Annunziata S., Mosconi M. (2021). Pulsed electromagnetic fields in bone healing: Molecular pathways and clinical applications. Int. J. Mol. Sci..

[128] Varani K., Vincenzi F., Ravani A., Pasquini S., Merighi S., Gessi S., Setti S., Cadossi M., Borea P.A., Cadossi R. (2017). Adenosine receptors as a biological pathway for the anti-inflammatory and beneficial effects of low frequency low energy pulsed electromagnetic fields. Mediat. Inflamm..

[129] Diller M.L., Master V. (2023). Integrative surgery: Embedding complementary and nonpharmacologic therapies into surgical pain management strategies. Am. Surg..

[130] Chen Q., Chen E., Qian X. (2021). A narrative review on perioperative pain management strategies in enhanced recovery pathways—The past, present and future. J. Clin. Med..

[131] Tan M., Law L.S.-C., Gan T.J. (2015). Optimizing pain management to facilitate enhanced recovery after surgery pathways. Can. J. Anesth..

[132] Wick E.C., Grant M.C., Wu C.L. (2017). Postoperative multimodal analgesia pain management with nonopioid analgesics and techniques: A review. JAMA Surg..

[133] Viderman D., Mukazhan D., Kapessova K., Tungushpayev M., Badenes R. (2024). The impact of ketamine on outcomes in acute pain management: An umbrella review. J. Clin. Med..

[134] Park C.M., Inouye S.K., Marcantonio E.R., Metzger E., Bateman B.T., Lie J.J., Lee S.B., Levin R., Kim D.H. (2022). Perioperative gabapentin use and in-hospital adverse clinical events among older adults after major surgery. JAMA Intern. Med..

[135] Martinez L., Ekman E., Nakhla N. (2019). Perioperative opioid-sparing strategies: Utility of conventional NSAIDs in adults. Clin. Ther..

[136] Chen Y., Wang E., Sites B.D., Cohen S.P. (2024). Integrating mechanistic-based and classification-based concepts into perioperative pain management: An educational guide for acute pain physicians. Reg. Anesth. Pain Med..

[137] Schreiber K.L., Wilson J.M., Chen Y.-Y.K. (2025). Recognizing pain phenotypes: Biopsychosocial sources of variability in the transition to chronic postsurgical pain. Reg. Anesth. Pain Med..

[138] Elhomsy P., Reliquet B., Besson G., Fawaz R. (2025). Radiofrequency and cryoneurolysis in pain management: Development, technique, and application. Pain Res. Manag..

[139] Pritzlaff S.G., Goree J.H., Hagedorn J.M., Lee D.W., Chapman K.B., Christiansen S., Dudas A., Escobar A., Gilligan C.J., Guirguis M. (2023). Pain education and knowledge (PEAK) consensus guidelines for neuromodulation: A proposal for standardization in fellowship and training programs. J. Pain Res..

[140] Orhurhu V., Hussain N., Karri J., Mariano E.R., Abd-Elsayed A. (2023). Perioperative and anesthetic considerations for the management of neuromodulation systems. Reg. Anesth. Pain Med..

[141] Morano J.M., Uejima J.L., Tung A., Rosenow J.M. (2023). Management strategies for patients with neurologic stimulators during nonneurologic surgery: An update and review. Curr. Opin. Anaesthesiol..

[142] Eissazade N., Rohani M., Fereshtehnejad S.-M., Sinaeefar M.J., Tabatabaee S. (2025). Potential interference of spinal cord and sacral stimulators with cardiac implantable electronic devices: A systematic review. Neurosurg. Rev..

[143] Dababou S., Marrocchio C., Scipione R., Erasmus H.-P., Ghanouni P., Anzidei M., Catalano C., Napoli A. (2018). High-intensity focused ultrasound for pain management in patients with cancer. Radiographics.

[144] Sowder T., Sayed D., Concannon T., Pew S.H., Strand N.H., Abd-Elsayed A., Wie C.S., Gomez Ramos D.E., Raslan A.M., Deer T.R. (2023). The American Society of Pain and Neuroscience (ASPN) guidelines for radiofrequency ablative procedures in patients with implanted devices. J. Pain Res..

[145] Johnson M.I. (2017). Transcutaneous electrical nerve stimulation (TENS) as an adjunct for pain management in perioperative settings: A critical review. Expert Rev. Neurother..

[146] Napoli A., De Maio A., Alfieri G., Gasperini C., Scipione R., Campanacci L., Siepe G., De Felice F., Siniscalchi B., Chiurchioni L. (2023). Focused ultrasound and external beam radiation therapy for painful bone metastases: A phase II clinical trial. Radiology.

[147] Viderman D., Nabidollayeva F., Aubakirova M., Sadir N., Tapinova K., Tankacheyev R., Abdildin Y.G. (2024). The impact of transcutaneous electrical nerve stimulation (TENS) on acute pain and other postoperative outcomes: A systematic review with meta-analysis. J. Clin. Med..

[148] Ilfeld B.M., Finneran J.J., Swisher M.W., Said E.T., Gabriel R.A., Sztain J.F., Khatibi B., Armani A., Trescot A., Donohue M.C. (2022). Preoperative ultrasound-guided percutaneous cryoneurolysis for the treatment of pain after mastectomy: A randomized, participant- and observer-masked, sham-controlled study. Anesthesiology.

[149] Cho A.M., Xiong J.S., Burns S.L. (2023). The emerging role of peripheral nerve stimulation in postoperative analgesia. Curr. Pain Headache Rep..

[150] Buzza A., Tapas K., Anders J., Jenkins M., Moffitt M. (2024). Photobiomodulation for pain relief: Model-based estimates of effective doses of light at the neural target. J. Photochem. Photobiol..

[151] Perez-Neri I., Gonzalez-Aguilar A., Sandoval H., Pineda C., Rios C. (2021). Therapeutic potential of ultrasound neuromodulation in decreasing neuropathic pain: Clinical and experimental evidence. Curr. Neuropharmacol..

[152] Asija S., Pahwa B., Agarwal A., Patil Y., Chaurasia B. (2024). Status of functional neurosurgery in lower middle-income countries (LMICs): A multinational cross-sectional survey-based analysis of exposure, utilization, and perceived barriers. Clin. Neurol. Neurosurg..

[153] Doshi P.P., Russo M., Doshi P.K. (2023). Practice trends of neuromodulation therapies for pain and spasticity in India. Neuromodulation Technol. Neural Interface.

[154] Lemoine A., Martinez V., Bonnet F. (2019). Pain measurement and critical review of analgesic trials: Pain scores, functional pain measurements, limits and bias of clinical trials. Best Pract. Res. Clin. Anaesthesiol..

[155] Pogatzki-Zahn E.M., Liedgens H., Hummelshoj L., Meissner W., Weinmann C., Treede R.-D., Vincent K., Zahn P., IMI-PainCare PROMPT Consensus Panel, Kaiser U. (2021). Developing consensus on core outcome domains for assessing effectiveness in perioperative pain management: Results of the PROMPT/IMI-PainCare Delphi meeting. Pain.

[156] Martini C.H., Boon M., Broens S.J.L., Hekkelman E.F., Oudhoff L.A., Buddeke A.W., Dahan A. (2015). Ability of the nociception level, a multiparameter composite of autonomic signals, to detect noxious stimuli during propofol–remifentanil anesthesia. Anesthesiology.

[157] Edry R., Recea V., Dikust Y., Sessler D.I. (2016). Preliminary intraoperative validation of the nociception level index: A noninvasive nociception monitor. Anesthesiology.

[158] Laferrièee-Langlois P., Morisson L., Jeffries S., Duclos C., Espitalier F., Richebe P. (2024). Depth of anesthesia and nociception monitoring: Current state and vision for 2050. Anesth. Analg..

[159] Ruetzler K., Montalvo M., Bakal O., Essber H., Rössler J., Mascha E.J., Han Y., Ramachandran M., Keebler A., Turan A. (2023). Nociception level index–guided intraoperative analgesia for improved postoperative recovery: A randomized trial. Anesth. Analg..

[160] Pavlov V.A., Chavan S.S., Tracey K.J. (2020). Bioelectronic medicine: From preclinical studies on the inflammatory reflex to new approaches in disease diagnosis and treatment. Cold Spring Harb. Perspect. Med..

[161] Falvey A., Metz C.N., Tracey K.J., Pavlov V.A. (2022). Peripheral nerve stimulation and immunity: The expanding opportunities for providing mechanistic insight and therapeutic intervention. Int. Immunol..

[162] Eberhardson M., Tarnawski L., Centa M., Olofsson P.S. (2020). Neural control of inflammation: Bioelectronic medicine in treatment of chronic inflammatory disease. Cold Spring Harb. Perspect. Med..

[163] Shiue S.-J., Rau R.-H., Shiue H.-S., Hung Y.-W., Li Z.-X., Yang K.D., Cheng J.-K. (2019). Mesenchymal stem cell exosomes as a cell-free therapy for nerve injury–induced pain in rats. Pain.

[164] Xu Y., Wang S., Li Z., Wang J., Wang M., Xue Q., Tao J., Huang S., Rastegar-Kashkooli Y., Xu Y. (2025). Bone marrow mesenchymal stem cell–derived exosomes alleviate neuropathic pain after spinal cord injury by inhibiting the TLR4/MyD88/NF-κB pathway. Exp. Neurol..

[165] Wei Z., Guo C., Zhou H., Wu Y., Zhou X., Chen J., Li F. (2025). Exosome-mediated miRNA delivery: A molecular switch for reshaping neuropathic pain therapy. Front. Mol. Neurosci..

[166] Joshi H.P., Jo H.-J., Kim Y.-H., An S.-B., Park C.-K., Han I. (2021). Stem cell therapy for modulating neuroinflammation in neuropathic pain. Int. J. Mol. Sci..

[167] Hwang J., Jang S., Kim C., Lee S., Jeong H.-S. (2023). Role of stem cell–derived exosomes and microRNAs in spinal cord injury. Int. J. Mol. Sci..

[168] Sajdeya R., Narouze S. (2024). Harnessing artificial intelligence for predicting and managing postoperative pain: A narrative literature review. Curr. Opin. Anaesthesiol..

[169] Mackey S., Aghaeepour N., Gaudillière B., Kao M.-C., Kaptan M., Lannon E., Pfyffer D., Weber K. (2025). Innovations in acute and chronic pain biomarkers: Enhancing diagnosis and personalized therapy. Reg. Anesth. Pain Med..

[170] Adams M.C.B., Bowness J.S., Nelson A.M., Hurley R.W., Narouze S. (2025). A roadmap for artificial intelligence in pain medicine: Current status, opportunities, and requirements. Curr. Opin. Anesthesiol..

[171] Harland T., Elliott T., Telkes I., Pilitsis J.G. (2024). Machine learning in pain neuromodulation. Adv. Exp. Med. Biol..

[172] Ciampi de Andrade D., García-Larrea L. (2023). Beyond trial-and-error: Individualizing therapeutic transcranial neuromodulation for chronic pain. Eur. J. Pain.

[173] Gupta A.K., Mena S., Jin Z., Gan T.J., Bergese S. (2021). Postoperative pain: A review of emerging therapeutic options. Expert Rev. Neurother..

[174] Mitra S., Carlyle D., Kodumudi G., Kodumudi V., Vadivelu N. (2018). New advances in acute postoperative pain management. Curr. Pain Headache Rep..

[175] Chen X., Thee C., Gruenewald M., Ilies C., Hocker J., Hanss R., Steinfath M., Bein B. (2012). Correlation of surgical pleth index with stress hormones during propofol–remifentanil anaesthesia. Sci. World J..

[176] Packiasabapathy S., Rangasamy V., Sadhasivam S. (2021). Pupillometry in perioperative medicine: A narrative review. Can. J. Anesth..

[177] Sabourdin N., Burey J., Tuffet S., Thomin A., Rousseau A., Al-Hawari M., Taconet C., Louvet N., Constant I. (2022). Analgesia Nociception Index–Guided Remifentanil versus Standard Care during Propofol Anesthesia: A Randomized Controlled Trial. J. Clin. Med..

